# Platelets Aggregate With Neutrophils and Promote Skin Pathology in Psoriasis

**DOI:** 10.3389/fimmu.2019.01867

**Published:** 2019-08-16

**Authors:** Franziska Herster, Zsofia Bittner, Marius Cosmin Codrea, Nathan K. Archer, Martin Heister, Markus W. Löffler, Simon Heumos, Joanna Wegner, Ramona Businger, Michael Schindler, David Stegner, Knut Schäkel, Stephan Grabbe, Kamran Ghoreschi, Lloyd S. Miller, Alexander N. R. Weber

**Affiliations:** ^1^Department of Immunology, University of Tübingen, Tübingen, Germany; ^2^Quantitative Biology Center, University of Tübingen, Tübingen, Germany; ^3^Department of Dermatology, Johns Hopkins University School of Medicine, Baltimore, MD, United States; ^4^Department of Dermatology, University Hospital Tübingen, Tübingen, Germany; ^5^Department of General, Visceral and Transplant Surgery, University Hospital Tübingen, Tübingen, Germany; ^6^Department of Clinical Pharmacology, University Hospital Tübingen, Tübingen, Germany; ^7^Department of Dermatology, University Hospital Mainz, Mainz, Germany; ^8^Division of Molecular Virology, Institute of Virology, Tübingen, Germany; ^9^Institute of Experimental Biomedicine, University Hospital and Rudolf Virchow Center, University of Würzburg, Würzburg, Germany; ^10^Department of Dermatology, University Hospital Heidelberg, Heidelberg, Germany; ^11^Department of Dermatology, Charité–Universitätsmedizin Berlin, Berlin, Germany

**Keywords:** psoriasis, neutrophil, platelet, platelet-neutrophil complexes (PNCs), imiquimod

## Abstract

Psoriasis is a frequent systemic inflammatory autoimmune disease characterized primarily by skin lesions with massive infiltration of leukocytes, but frequently also presents with cardiovascular comorbidities. Especially polymorphonuclear neutrophils (PMNs) abundantly infiltrate psoriatic skin but the cues that prompt PMNs to home to the skin are not well-defined. To identify PMN surface receptors that may explain PMN skin homing in psoriasis patients, we screened 332 surface antigens on primary human blood PMNs from healthy donors and psoriasis patients. We identified platelet surface antigens as a defining feature of psoriasis PMNs, due to a significantly increased aggregation of neutrophils and platelets in the blood of psoriasis patients. Similarly, in the imiquimod-induced experimental *in vivo* mouse model of psoriasis, disease induction promoted PMN-platelet aggregate formation. In psoriasis patients, disease incidence directly correlated with blood platelet counts and platelets were detected in direct contact with PMNs in psoriatic but not healthy skin. Importantly, depletion of circulating platelets in mice *in vivo* ameliorated disease severity significantly, indicating that both PMNs and platelets may be relevant for psoriasis pathology and disease severity.

## Key Points

- Neutrophils in the blood of psoriasis patients show a distinct “platelet signature” of surface antigens.
- Platelets congregate with neutrophils in psoriatic skin lesions.
- Circulating platelets contribute to psoriasis skin pathology.

## Introduction

Psoriasis is a frequent, chronic, immune-mediated inflammatory skin disease of unknown etiology (1). Its most common form, plaque psoriasis, shows epidermal hyperplasia, increased endothelial proliferation, and a prominent infiltrate of polymorphonuclear neutrophils (PMNs) (1, 2). The accumulation of PMNs in psoriatic plaques and micro-abscesses is accompanied by an increase of PMNs in the circulation of psoriasis patients but their precise role in the disease remains enigmatic (2, 3). Furthermore, it remains unclear which factors prompt PMNs, plasmacytoid dendritic cells and T cells to accumulate in psoriatic skin. The latter cells drive a chronic phase of disease, dominated by IL-17 cytokines (1, 2, 4). Apart from its strong manifestation in the skin, psoriasis is now considered a systemic disease, and besides frequent joint involvement (psoriasis arthritis), alterations in circulating immune cell subsets have been reported: for example, the frequency of CD16^+^ monocytes is altered in psoriasis patients and these cells were observed to aggregate increasingly with other monocytes or lymphocytes in patient blood (5). Changes within the T cell compartment have also been reported (6). Additionally, a strong link between psoriasis and cardiovascular comorbidities has been noted (7–9), for which IL-17A may be an important factor (10). Regarding cardiovascular events, psoriasis severity correlates with the incidence of cerebrovascular, peripheral vascular and heart structural disorders [reviewed in (7)]. Here a potential etiology involving platelets has been proposed (11) but was not experimentally proven, especially regarding a direct link between PMNs and platelets in humans.

Recent research has uncovered an intimate relationship between different leukocyte populations, including PMNs, and platelets. Interestingly, the existence of direct leukocyte-platelet aggregates *in vitro* and in human blood has been known for some time (12, 13). Ludwig et al. reported that P-selectin Glycoprotein Ligand-1 (PSGL-1)-P-selectin-mediated interactions between platelets and leukocytes promoted rolling in murine skin micro vessels and the same receptors were responsible for activated platelets to interact with murine PBMCs, when co-cultured *in vitro* (14). When these co-cultured PBMCs were infused in mice, rolling in the murine skin microvasculature was observed. The authors speculated whether platelets might also prompt leukocyte invasion into the inflamed skin and noted that P-selectin expression correlated with psoriasis score. However, their studies were largely based on transfusion experiments and did not focus on PMNs. More recently, the capacity of platelets to direct PMN extravasation experimentally was extensively studied by *in vivo* models (15), and (16) proposed that initially CD40-CD40L interactions mediate leukocyte capture at the vessel wall for PSGL-1-P-selectin interactions to guide subsequent extravasation. Whilst this is required for peripheral defense, in rheumatoid arthritis it was also observed that platelets attract neutrophils into the synovium where they become trapped and contribute to disease severity (17). Whether Platelet-Neutrophil complexes (PNCs) occur in human psoriasis patients and whether they contribute to psoriasis skin inflammation was not experimentally studied.

In order to identify surface antigens on human peripheral blood immune cells that might be involved in their skin-homing in psoriasis, we screened 322 surface antigens in PMNs, monocytes and T- and B-lymphocytes from psoriasis patients and healthy controls. This unbiased approach identified surface antigen signatures specific for different blood immune cell populations in psoriasis. For PMNs platelet markers were significantly increased and this surface antigen signature was attributable to direct PNCs. Such PNCs were also observed upon psoriasis induction in mice and in the skin of psoriasis patients. Interestingly, depletion of platelets in the imiquimod (IMQ)-induced experimental *in vivo* setting of psoriasis drastically decreased ear and epidermal thickness of the skin. Collectively, our results establish a functional link between circulating PMN-platelet aggregates and disease activity in the psoriatic skin.

## Materials and Methods

### Reagents

All chemicals were from Sigma or Invitrogen, respectively, unless otherwise stated in Supplementary Materials. Antibodies and recombinant cytokines are listed in Table S1.

### Mice

Sex- and age-matched C57Bl/6J SPF mice (Jackson Laboratories) bred and maintained according to local animal welfare guidelines and applicable regulations (see Supplementary Information) were used at the age of 6–8 weeks. Mice were bred and maintained under the same specific pathogen-free conditions at an American Association for the Accreditation of Laboratory Animal Care (AAALAC)-accredited animal facility at Johns Hopkins University and handled according to procedures described in the Guide for the Care and Use of Laboratory Animals as well as Johns Hopkins University's policies and procedures as set forth in the Johns Hopkins University Animal Care and Use Training Manual, and all animal experiments were approved by the Johns Hopkins University Animal Care and Use Committee.

### Study Participants and Sample Acquisition

All patients and healthy blood donors included in this study provided their written informed consent before study participation. Approval for use of their biomaterials was obtained by the local ethics committees at the University Hospitals of Tübingen and Heidelberg, in accordance with the principles laid down in the Declaration of Helsinki as well as applicable laws and regulations. All blood or skin samples obtained from psoriasis patients (median age 41.8 years, psoriasis area severity index (PASI) ≥10 (except for two donors in **Figures 2A,B**, Figures S3B,C where PASI was >4.5) no systemic treatments at the time of blood/skin sampling) were obtained at the University Hospitals Tübingen or Heidelberg, Departments of Dermatology, and were processed simultaneously with samples from at least one healthy donor matched for age and sex (recruited at the University of Tübingen, Department of Immunology). Skin sections were obtained from 11 patients with plaque psoriasis and one patient with psoriasis guttata. Platelet counts were determined in the course of established clinical routines at the time of study blood sampling.

### Cell Surface Marker Expression Screening in Whole Blood

**Figure 1:**
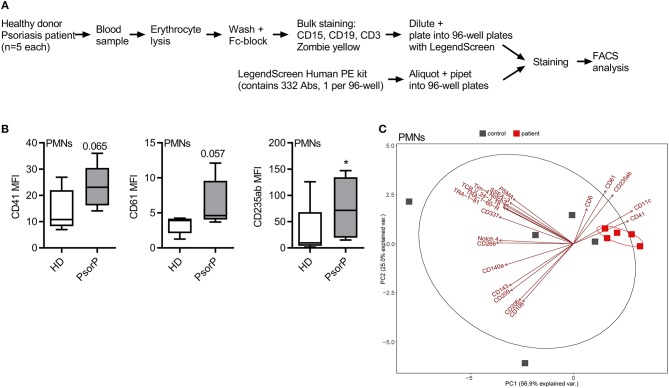
Psoriasis PMNs show a distinct “platelet signature.” **(A)** Schematic overview of surface antigen screen procedure. **(B)** Selected surface antigens with the most pronounced differences in MFI between healthy blood donors (HDs) and psoriasis patients (PsorP) PMNs, *n* = 5 each. **(C)** Principal component analyses of PMN surface antigens for healthy donors (red) and psoriasis patients (gray) with top significant antigens (based on nominal *p* < 0.1, *n* = 5 each) contributing to a separation of patients and healthy controls. **(B,C)** represents combined data (mean + SD) from “*n*” biological replicates (each dot represents one donor). *p < 0.1 nominal by two-way ANOVA followed by Tukey's multiple comparisons correction.

A cell surface antigen screening was performed using the LegendScreen from Biolegend (Figure 1A). Whole blood (EDTA-anticoagulated) was drawn from five psoriasis patients and five sex- and age-matched controls. Erythrocyte lysis was performed for 5 min at 4°C on a roller shaker using 154 mM NH_4_Cl, 10 mM KHCO_3_, 0.1 mM EDTA pH 8 (10× buffer), pH of buffer adjusted to 7.3 and sterile filtered (0.22 μm). After a short spin, FC block was performed and the cells were stained with anti-CD3, -CD15, and -CD19, excluding dead cells using Zombie Yellow. Subsequently, the stained cells were aliquoted into 96 well-plates, each containing a PE-labeled antibody directed against one of 332 surface antigens, and 10 isotype controls in PE. The following washing and further steps were performed using the manufacturer's instructions, except that one kit was divided for the measurement of four donors. FACS measurements were performed using a MACSQuant analyzer (Miltenyi) and subsequently FlowJo V10 was used to analyze the data. The gating strategy is depicted in Figure S1 and T cells, PMNs, and B cells gated according to the Abs in the master mix. Monocytes were gated by granularity and size but not additionally verified with CD14 staining. However, in the well-containing anti-CD14-PE Abs, all gated events were CD14-positive.

### Differential Expression Analysis of Surface Marker Screening Data

Conceptually, the goal was to identify surface antigens, which are significantly different between patients and healthy donors within the targeted cell types. This relationship was formulated as MFI ~ health_status + cell_type, where the mean fluorescence intensity (MFI) depends on the two main factors: health_status with levels {patient, healthy donor} and cell_type with levels {B cells, Monocytes, Neutrophils, T cells} [(16) T cells], gated as indicated in Figure S1. All cell types were measured simultaneously in one FACS screen per subject (patient or healthy donor) and the resulting intrinsic within-subject variance (across cell types) was accounted for by extending MFI ~ health_status + cell_type + subject/cell_type. In this notation, cell_type is “nested” within “subject,” accommodating the above. The implementation of the analysis was done in R [version 3.4.4, with linear mixed models using the R package nlme (18, 19) (version 3.1-131.1, details and code upon request). The fitted models were subject to *post-hoc* analysis with Tukey's “Honest Significant Difference” test to compute adjusted pair-wise differences among the cell types (20). The lsmeans (version 2.27-61) (21) R package implementation was used in order to compute the adjustments. With lsmeans' pairs all pair-wise contrasts of patient vs. healthy donor by the given cell_type were calculated. From all contrasts of the different cell_type levels the *p*-values and the fold changes of all surface antigens were extracted. This analysis provides nominal, multiple-comparison-adjusted *p*-values and fold changes between patients vs. healthy donors for each cell type (20). In this exploratory setting, surface antigens with *p* < 0.1 were included into the combined analysis. For the platelet analysis an unpaired *t*-test method was used as only one cell type had to be considered. Receptors with MFI values missing for more than one donor per group were filtered out. For the generation of the PCA plots, the R package ggplot2 (version 2.2.1) was used.

### Flow Cytometry

Two hundred microliter of the cell suspension was transferred into a 96 well-round bottom plate and spun down for 5 min at 448 × g, 4°C. FcR block was performed using pooled human serum diluted 1:10 in FACS buffer (PBS, 1 mM EDTA, 2% FBS heat inactivated) for 15 min at 4°C. After washing, the samples were stained for 20–30 min at 4°C in the dark. Thereafter, fixation buffer (4% PFA in PBS) was added to the cell pellets and incubated for 10 min at RT in the dark. After an additional washing step, the cell pellets were resuspended in 100 μl FACS buffer. Measurements were performed on a MACSQuant analyzer (Miltenyi). Analysis was performed using FlowJo V10 analysis software.

### Fluorescence Microscopy of Fixed Whole Blood Cells

The cells were seeded in a 96 well-plate, 200 μl cell suspension per well. FcR block, staining, fixation, and permeabilization were performed as for flow cytometry. After 0.05% Saponin permeabilization, nuclear DNA was stained using Hoechst 33342 (1 μg/ml, Thermo Fisher). The cell pellets were resuspended in 50–100 μl FACS buffer. Forty microliter of the cell suspension was pipetted on a Poly-L-Lysine coated coverslip (734–1005, Corning) and the cells were left to attach for 1 h in the dark. ProLong Diamond Antifade (P36965, Thermo Fisher) was used to mount the coverslips on uncoated microscopy slides. The slides were left to dry overnight at RT in the dark and were then stored at 4°C before microscopy. The measurements were conducted with a Nikon Ti_2_ eclipse (100× magnification) and the analysis was performed using ImageJ/Fiji analysis software (22).

### Fluorescence Microscopy of Tissue Samples (Human and Mouse)

Skin samples from psoriasis patients or healthy skin samples and mouse ear samples were paraffin-embedded according to standard procedures and were then deparaffinized and rehydrated using Roti Histol (Roth, 6640.1) and decreasing concentrations of ethanol (100 (2×), 95, 80, and 70%). After rinsing in ddH_2_O, antigen retrieval was performed by boiling for 10–20 min in citrate buffer (0.1 M, pH = 6). The skin tissue was then washed three times for 5 min with PBS. Blocking was performed using pooled human serum (1:10 in PBS) for 30 min at RT. The primary antibody was added either overnight at 4°C or for 1 h at RT. After three washes, the secondary antibody was incubated for 30 min at RT in the dark. Thereafter, the samples were washed again and Hoechst 33342 (1 μg/ml, ThermoFisher) was added for 5 min. Then three last washes were performed before using ProLong Diamond Antifade (P36965, Thermo Fisher) for mounting. The samples were left to dry overnight at RT in the dark before being used for microscopy or stored at 4°C. The specimens were analyzed on a Nikon Ti2 eclipse microscope (40–60× magnification) and the analysis was performed using ImageJ/Fiji analysis software.

### Imiquimod Model of Psoriatic Skin Inflammation

Prior to and after daily topical application of 5% imiquimod cream to the ears of anesthetized (2% isoflurane) mice ear thickness was measured with a manual caliper (Peacock) on d0 to d5. For flow cytometry analysis, retro-orbital blood samples were collected on d0 and d5, diluted in TBS containing 5 U/ml Heparin and subsequently PBS and an aliquot stained using appropriate antibodies (see Table S1). Murine blood PMNs were detected by CD11b + Ly6C^int^ cells, thereby avoiding issues with antibody masking by anti-Ly6G (mAb clone 1A8). The same applied to murine blood platelets, which were depleted with anti-CD42b mAb, but detected by flow cytometry with anti-CD41 mAbs. Full thickness ear skin was excised on d5, fixed in 10% formalin and paraffin-embedded. Skin cross-sections (4 μm) were stained by H & E according to standard procedures. At least 10 epidermal thickness measurements per mouse were averaged.

### PMN or Platelet Depletion and PSGL-1 Blockade

For PMN depletion, 500 μg of anti-Ly6G mAb (clone 1A8) or isotype control were diluted in sterile PBS and injected i.p. on days−2, 0, 2, and 4 of IMQ treatment. For platelet depletion in mice, 4 μg/g of anti-CD42b (clone R300, Emfret Analytics) or rat IgG isotype control (clone R301, Emfret Analytics) in sterile PBS was administered i.v. one day before, and 2 μg/g administered i.p. 3 days after the first IMQ treatment. For PSGL-1 blockade, 50 μg anti-PSGL-1 or Rat IgG1 isotype control (both BioXcell) were diluted in sterile PBS and injected i.v. on day−1, and again i.p. on d1 and d3 of IMQ treatment.

### Statistics

Statistics regarding differential surface antigen expression analysis are described above. All other experimental data were analyzed using Excel 2010 (Microsoft) and/or GraphPad Prism 6, 7, or 8, microscopy data with ImageJ/Fiji, flow cytometry data with FlowJo V10. In case of extreme values, outliers were statistically identified using the ROUT method at a stringency of 2% and normality tested using the Shapiro-Wilk test for the subsequent choice of a parametric or non-parametric test. *p*-values (α = 0.05, β = 0.8) were then calculated and multiple testing was corrected for in Prism, as indicated in the figure legends. Values < 0.05 were considered as statistically significant and denoted by ^*^ throughout. Comparisons were made to unstimulated controls unless indicated otherwise by brackets.

## Results

### Circulating Neutrophils in Psoriasis Show a Distinct Platelet Signature of Surface Antigens

To phenotype immune cells in psoriasis patients with a view to identifying surface antigens disparately regulated in psoriasis PMNs, we combined the so-called LegendScreen, a screening format consisting of antibodies directed against *n* = 332 different surface antigens and 10 corresponding isotype controls, with a whole blood staining assay using anti-CD3, CD15, CD19 Abs and a live/dead marker (Figure 1A), based on previously published work (23). Five psoriasis patients with PASI ≥ 10 and without systemic therapy (at the time of blood drawing) and 5 healthy donors were analyzed, the major cell populations gated as described in Figure S1A and mean fluorescence intensities (MFIs) acquired for each of the 322 surface antigens (Table S2). Differential expression analysis was performed by two-way ANOVA followed by Tukey's multiple comparisons correction (see section Methods and Supplemental R code). Conceptually, the goal of the differential expression analysis was to identify surface antigens, which are significantly different between PMNs from patients and healthy donors. Figure 1B shows selected surface antigens with the most prominent MFIs differences between patient and control PMNs. Evidently, monocytes also showed multiple differentially regulated surface antigens (Table S1 and Figure S2). Although individual surface markers were of primary interest, we next considered whether a certain combination (“signature”) of molecules was suitable to discriminate patients and healthy donors. In order to check whether the most differently expressed surface antigens (based on a nominal *p* < 0.1) were able to separate the two groups a principal component analysis (PCA) was performed (Figure 1C). This showed that for PMNs from psoriasis patient samples clustered together tightly (Figure 1C). Antigens contributing significantly to the separation of both groups were CD6, CD11c, CD41, CD61, and CD235ab. As CD6 expression was not detectable on PMNs from psoriasis patients or from healthy controls in follow-up flow cytometry, CD6 was considered as false positive and not pursued any further. CD11c is a known marker on DCs, monocytes but also granulocytes (24) and is considered to be important for phagocytosis and for DC antigen presentation (25, 26). It is conceivable that CD11c is also found on PMNs and may displays similar functions there. CD235ab expression is usually found on terminally differentiated, anucleated erythrocytes and considered to be erythrocyte-specific (27). We detected higher CD235ab expression on PMNs from psoriasis patients. As PMNs can become more “sticky” under certain conditions, e.g., in cancer (28), we interpreted CD235ab positivity to likely result from membrane-fragments of erythrocytes stuck to PMNs. Notably, two of the five combination markers, namely CD41 (also known as GPIIb/IIIa) and CD61 (also termed Integrin β3), are known platelet antigens, which usually act in concert and are required for platelet adhesion and aggregation (29, 30). As little is known regarding the role of these molecules in psoriasis, we focused on the surface antigens CD41 and CD61 in the following. Collectively, our analysis in untreated whole blood samples shows significant differences in surface antigen expression across circulating immune cell populations and that selected groups of surface antigens are able to discriminate patients and healthy donors on the basis of PMN surface antigens.

### Circulating Neutrophils in Psoriasis Directly Interact With Platelets

**Figure 2:**
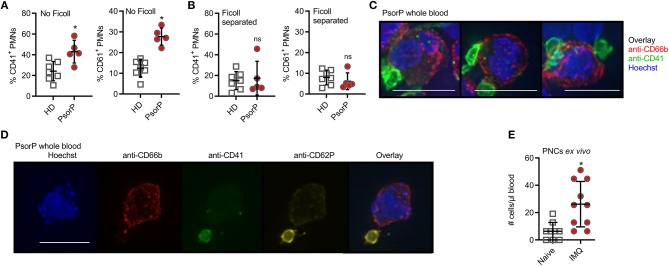
Psoriasis PMNs directly interact with platelets more prominent in psoriasis blood samples and upon *in vivo* psoriasis induction. **(A,B)** Flow cytometric analysis of CD41- **(A)** or CD61-positive **(B)** PMNs (defined as CD15^+^CD66b^+^ and CD41^+^ or CD61^+^) analyzed in HD or PsorP (HD *n* = 7, PsorP *n* = 5) in whole blood samples (no Ficoll centrifugation, left panels) or Ficoll density gradient centrifugation (right panels). **(C)** Fluorescence microscopy of PMNs in a PsorP whole blood stained as indicated (scale bar = 10 μm). **(D)** as in **(C)**. **(E)** Mean number of PMN-platelet aggregates comparing naïve (day 0) and IMQ-treated (day 5) isotype mice (*n* = 10 each). **(A,B,E)** represent combined data (mean + SD) from “*n*” biological replicates (in **A,B,E** each dot represents one donor or mouse). In **(C,D)** one representative of “*n*” biological replicates (donors) is shown (mean + SD). *p < 0.05 according to Mann-Whitney test **(A,B)**, unpaired Student's *t*-test **(E)**.

Since PMN infiltration is a hallmark of psoriasis, we focused on PMN surface antigens and noted that several of the aforementioned psoriasis-associated surface antigens, e.g., CD41 and CD61, are typically associated with platelets. These data indicated a “platelet-like” signature on psoriasis PMNs, and, upon re-inspection of the screen data, CD41 and CD61 showed pronounced differences for PMNs that barely missed statistical significance (*cf*. Figure 1B). As mentioned before, platelet association with PMNs in the form of PNCs is not uncommon, especially in the context of inflammatory disorders. Whereas, PNCs would persist in unmanipulated whole blood, Ficoll density gradient purification was shown to separate PMNs and platelets due to their different densities and hence sedimentation behavior (31). In order to check whether increased platelet marker expression on PMNs was due to *de-novo* surface expression on PMNs or due to platelet association to PMNs, we analyzed whole blood and Ficoll-purified PMNs together with PBMCs from the same patients and healthy controls that were processed identically. Indeed, compared to whole blood staining, where psoriasis patient samples showed higher CD41 and CD61 expression than healthy donors (Figure 2A), Ficoll purification reduced these differences in CD41 and CD61 staining between psoriasis patients and healthy donors: Ficoll-purified PMNs showed more similar levels of CD41 and CD61 positivity between the two groups (Figure 2B). As Ficoll is unlikely to lead to a shedding of CD41 or CD61 (for example, CD62L was unaffected), we concluded that the platelet surface markers seen in psoriasis PMNs in whole blood analysis were due to formation of PNCs. Similar results were obtained for monocytes, which also formed complexes with platelets (**Figures S3A–C**). The existence of PNCs was confirmed by bright-field fluorescence microscopy, which showed small CD41^+^ CD62P^+^ entities, clearly interpretable as platelets, attached to the outer surface of the CD66b^+^ PMNs (Figure 2C). Including anti-CD62P (P-Selectin) Abs in the analysis, we noted that the platelets were CD62P^+^ (Figure 2D) i.e., activated, in line with recent analyses (15). Thus, the expression of platelet markers on psoriasis PMNs is attributable to an increased formation of PNCs.

To gain deeper insight into whether the formation of PNCs and monocyte-platelet complexes may be a result of skin inflammation, we analyzed complex formation in the IMQ-induced *in vivo* mouse model of experimental psoriasis. In this model the application of the TLR7 agonist IMQ leads to a fulminant local and psoriasiform inflammation accompanied by PMN influx (32). Figure 2E shows that IMQ-treated mice exhibited significantly higher levels of PNCs compared to control mice, in agreement with the data obtained from humans with psoriasis. Monocyte-platelet aggregates were also affected (Figure S3D). Collectively, these results indicate that PNCs and monocyte-platelet complexes are increased in psoriasis and co-incide with skin inflammation.

### Platelets Are Detectable in Psoriatic Skin and Show Distinct Surface Antigens in the Circulation of Psoriasis Patients

**Figure 3:**
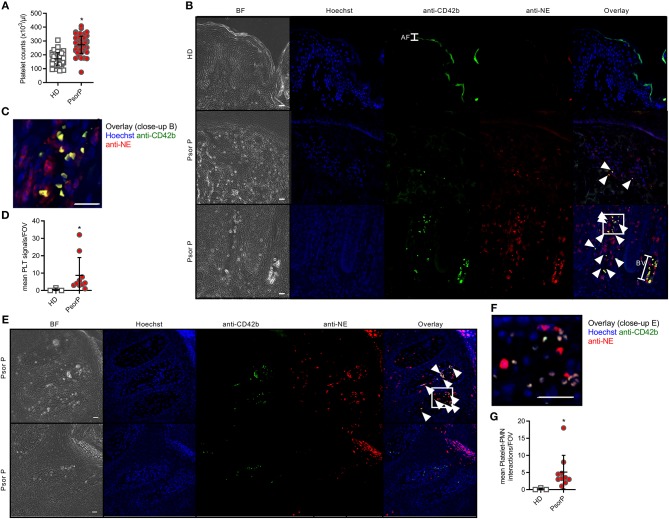
Platelets are more abundant in the blood and skin of psoriasis patients. Total platelet counts in HDs and PsorP (**A**, *n* = 52 vs. 53). **(B,C)** Fluorescence microscopy analysis of skin sections stained as indicated from healthy donor or psoriasis-affected skin (*n* = 12 patients and three healthy controls, scale bar = 20 μm, close-up shown in **C**) and quantified in **(D)**. **(E,F)** as in **(B)** but showing platelet-PMN aggregates in certain skin samples (close-up shown in **F**), quantified in **(G)**. AF, autofluorescence; BV, blood vessel. **(A,D,G)** represent combined data (mean + SD) from “*n*” biological replicates (each dot represents one donor or mouse). In **(B,C,E,F)** representatives of “*n*” biological replicates (donors) are shown (mean + SD). *p < 0.05 according to an unpaired Student's *t*-test **(A)** or Mann-Whitney test **(C,E)**.

Although platelet activation is known to promote leukocyte association, we wondered whether a mere increase in total platelet counts would correlate with psoriasis, as an increased platelet count might favor platelet-PMN association. As expected, platelet counts in the circulation of psoriasis patients were 62% higher than in healthy blood donors (*p* < 0.0001, Figure 3A). To investigate if this higher abundance might also pertain to the skin of patients where PMNs are frequently infiltrating, we stained for platelet markers in psoriasis skin, which according to our knowledge has not been done before. Whereas, neither PMNs (identified by anti-neutrophil elastase, NE, Abs) nor platelets (stained using anti-CD41 and –CD42b) were readily detectable in healthy skin or isotype-stained samples, in psoriasis skin samples both cell types were present (Figures 3B,C and quantified in Figure 3D). Certain samples even showed co-localization of PMNs and platelets within infiltrated areas (Figures 3E,F and quantified in Figure 3G). This indicates that platelets are only infiltrating lesional skin, and may preferentially do so in tandem with PMNs.

### Interference With Platelets *in vivo* Ameliorates Skin Pathology

**Figure 4:**
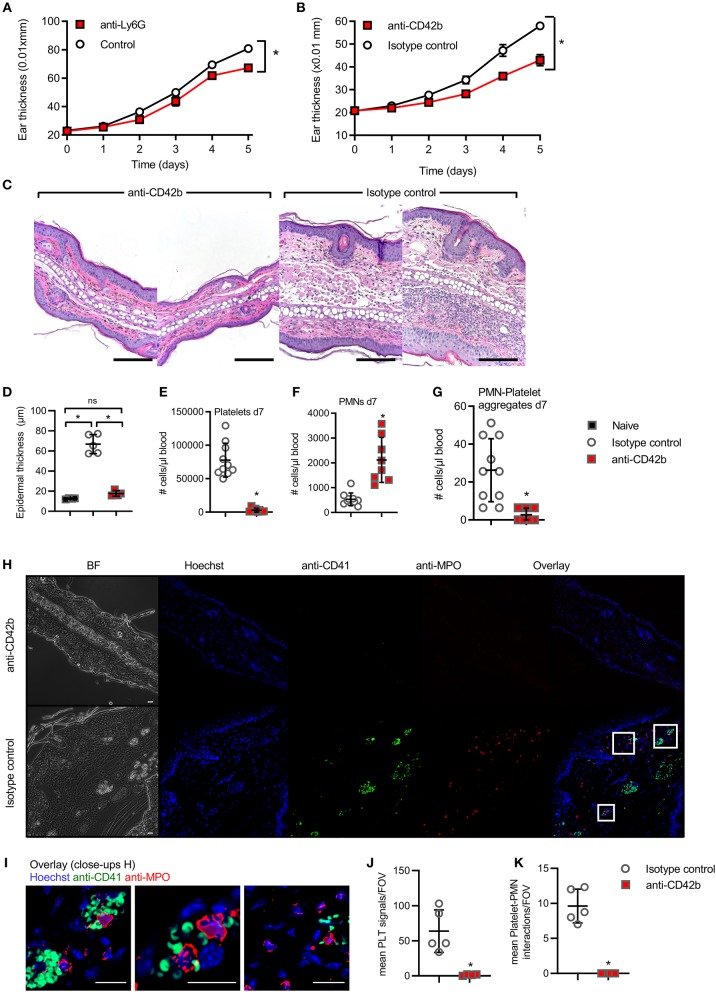
*In vivo* interference with platelets in experimental psoriasis model. **(A,B)** IMQ-induced experimental psoriasis model in BL/6 mice with ear thickness (mm × 0.01) measured upon PMN depletion by anti-Ly6G **(A)**, platelet depletion by anti-CD42b **(B)**, **(C)** representative H&E staining from anti-CD42b Ab and isotype-treated skin (*n* = 4–5, scale bar = 180 μm), quantified in **(D)**. **(E,G)** Flow cytometry analysis of total platelet counts on d7 **(E)**, PMN-platelet **(F)** and free PMNs **(G)** on d7 upon infusion of IMQ-treated animals with anti-CD42b Ab or isotype control (*n* = 7–8 each). **(H,I)** representative IF staining from anti-CD41 and isotype-treated skin (*n* = 4–5 for each group, scale bar = 20 μm, close-ups shown in **I**) and the quantification of platelet aggregates in the skin **(J)** and platelet-PMN aggregates **(K)** is shown. **(A,B,D–G,J,K)** represent combined data (mean + SD) from “*n*” biological replicates (each dot represents one mouse). In **(C,H,I)** representatives of “*n*” biological replicates (mouse biopsies) is shown *p < 0.05 according to two-way ANOVA **(A,B)**, unpaired Student's *t*-test **(D,I,J)**, Mann-Whitney test **(E–G)**.

Consistent with previous results (33), PMNs are relevant for disease severity in the IMQ-induced psoriasis model (Figure 4A, significant depletion verified in Figure S4A), as shown using anti-Ly6G Abs that were shown to both deplete and block the recruitment of PMNs (34). To check whether platelets are also causally involved in PMN skin accumulation and aggravate skin pathology in the context of psoriasis, we investigated the effects of platelet depletion in the IMQ model. Importantly, *in vivo* depletion of circulating platelets on d1 and d3 of IMQ-treatment using anti-CD42 antibodies (35), but not control IgG, showed a significant decrease in ear skin thickness (Figure 4B) and epidermal dysplasia (Figure 4C, quantified in Figure 4D), concomitant with the verified depletion of total platelets in circulation (Figure 4E). In accordance with the decrease in PMN-platelet aggregates (Figure 4F), the number of free PMN was significantly increased in the blood (Figure 4G). Moreover, platelets, often in close proximity to PMNs, were frequently found in IMQ-isotype treated mouse skin (Figure 4H), positive for anti-Histone H3 extracellular staining, indicating the possibility for platelets to activate PMNs and assist in NET formation (36) (Figure S4C). Conversely, platelet-depleted mice showed no PMN or platelet infiltration (Figures 4H,I and quantified in Figures 4J,K). When PSGL-1 (PMNs)-P-selectin (platelets) interactions were probed using i.v. infusion of anti-PSGL-1 Abs (14, 15) in a preliminary experiment, we observed an intermittent and intermediate effect on ear thickness compared to control IgG (Figure S4B). Taken together, these data indicate that circulating platelets significantly contribute to IMQ-induced skin inflammation and pathology.

## Discussion

In this present study, we screened the surface antigens of different immune cells in the blood of psoriasis patients and identified respective alterations compared to healthy controls, most notably an association of PMNs with platelets. Although the power of our analysis is limited in terms of sample size and by only nominal *p*-values calculated for individual surface antigens, PCA analysis showed different combinations of surface receptors to distinguish patients from healthy donors. It will be informative to analyze the suggested surface antigen combinations in larger cohorts and in the course of different topical or systemic psoriasis treatments. The whole blood-based protocol developed here may be useful for such a future application.

Psoriasis has long been viewed as an immunologically-driven disease, in which different leukocytes and T cells play important roles. Although platelet interactions with leukocytes have been reported for psoriasis mouse models and are typically found in many other inflammatory diseases (11, 14, 37), a specific role for platelets in the skin lesions of psoriasis patients has not been noted. In agreement with the systemic inflammatory nature of psoriasis, we found platelet-PMN (and –monocyte) aggregates in the circulation to correlate with skin disease. Of note, the depletion of circulating platelets—and consequently aggregates thereof–unexpectedly and drastically ameliorated skin disease. Our data are in line with platelet depletion experiments conducted in an experimental model of atopic dermatitis, another inflammatory skin disease characterized by severe itching of the skin (38). For both atopic dermatitis and psoriasis it could be envisaged that extravasation of leukocytes from skin-proximal blood vessels is facilitated by platelets and platelets then are transported along into the skin. There are several possible underlying mechanisms that await exploration: for example, serotonin-mediated increased vascular permeability induced by platelets (39) may contribute to the effect on PMN infiltration, a notion that fits with the observation that serotonin uptake inhibitors reduce the need for systemic therapies in psoriasis (40). Alternatively, platelets could directly steer the infiltration of their PMN complex partners. The small and intermittent effect of anti-PSGL1 treatment (*cf*. Figure S4B) would be consistent with such a notion: Ludwig and colleagues indeed showed that PSGL-1-P-selectin-mediated direct interactions between platelets and leukocytes promoted rolling in murine skin micro vessels (14). Alternative mediators, such as peripheral node addressin (PNAd) and neutrophil L-selectin (41) may explain the only partial effects of PSGL-1 blockade. Whether a scenario of joint PNC “piggyback homing” applies only to the skin or whether other organs apart from the skin, e.g., aortic tissue, are also affected remains to be investigated but may unearth a possible link to the cardiovascular comorbidities observed in psoriasis patients (10). Whichever the mechanism, it is conceivable that platelets that end up in the skin could directly contribute to disease severity—either in concert with leukocytes (42, 43) or acting separately. The responsiveness of platelets to microbe- and danger-associated molecular patterns, the ability for active locomotion and for secretion of inflammatory mediators described for platelets may be properties that could be involved (11, 44). Although a feedback to myelopoiesis cannot be excluded, the increase of free PMNs in the blood of platelet-depleted mice may suggest that the absence of circulating platelets traps PMNs in circulation and prevents them from entering and fueling local inflammation in the skin. If so, this would add psoriasis to the list of pathologies in which therapeutics preventing platelet-PMN interactions may prove beneficial but possibly at the expense of reducing peripheral innate defenses, which rely on PMNs being able to enter the periphery (15).

Platelets have been credited with the ability to promote peripheral homing of innate immune cells. However, the observation that platelets themselves enter psoriatic skin was unexpected. Further exploration of the role of skin-infiltrating platelets and their mechanisms of skin-homing or even active migration (45) in psoriasis and other inflammatory conditions may thus prove informative. On the other hand, the role of platelets in the cardiovascular comorbidities often associated with psoriasis is intriguing. Given the sensitivity of platelets to IL-17 (46), a key psoriasis-associated cytokine, investigation of the IL-17-axis and effects of anti-IL-17 biologicals on isolated platelets and platelet-leukocyte aggregates may prove insightful in this context.

## Ethics Statement

This study was carried out in accordance with the recommendations of the local ethics committees at the University Hospitals of Tübingen and Heidelberg, as well as the principles laid down in the Declaration of Helsinki and applicable laws and regulations. Approval for biomaterial use was obtained from the local ethics committees. All included subjects provided their written informed consent.

## Author Contributions

FH, ZB, and NA performed experiments. AW, FH, MC, NA, and SH analyzed data. MH, ML, JW, RB, MS, DS, KS, SG, KG, and LM were involved in sample and reagent acquisition. FH and AW were involved in the conceptual development of the study and wrote the manuscript. AW co-ordinated and supervised the entire study. All authors commented on or revised the manuscript.

### Conflict of Interest Statement

The authors declare that the research was conducted in the absence of any commercial or financial relationships that could be construed as a potential conflict of interest.

## Supplementary Material

Figure S1 Gating strategy used in the surface antigen screen in human whole blood.

Figure S2 Selected surface antigens with significant differences in MFI between the monocytes from healthy donors (HDs) and psoriasis patients (PsorP), *n* = 5 each. Graphs represent combined data (mean+SD) from ‘n' biological replicates. ^*^*p* < 0.1 nominal by two-way ANOVA followed by Tukey's multiple comparisons correction.

Figure S3 **(A)** Expression analysis screening results for CD41 (upper) and CD61 (lower panel) MFIs on gated monocytes between healthy blood donors (HDs) and psoriasis patients (PsorP), *n* = 5 each. **(B,C)** Flow cytometric analysis of CD41- or CD61-positive gated monocytes in HD or PsorP (HD *n* = 7, PsorP *n* = 5) in whole blood samples **(B)** or Ficoll density gradient centrifugation **(C)**. **(D)** Mean number of monocyte-platelet aggregates comparing naïve (d0) and IMQ-treated (d5) isotype mice (*n* = 10 each). **(A–D)** Represent combined data (mean+SD) from “n” biological replicates (each dot represents one donor). ^*^*p* < 0.1 nominal by two-way ANOVA in **(A–D)** ^*^*p* < 0.05 by unpaired Student's t-tests.

Figure S4 **(A)** Flow cytometry analysis of PMN depletion on d5 upon infusion of IMQ-treated animals with anti-Ly6G Abs or isotype control (*n* = 9–10 each). The data represent cells first gated on live cells with PI, then gated on CD11b+ cells, and the data represent Ly6Cint cells as percentage of the CD11b+ population. Cells were isolated from blood at the end of the experiment for both isotype control and anti-Ly6G (clone 1A8) treated mice. **(B)** Ear thickness (mm × 0.01) measured upon anti-PSGL-1 or control antibody infusion (*n* = 4–5 each). **(C)** Representative IF staining from IMQ-treated mouse ear skin (*n* = 5, scale bar = 20 μm). P, platelet; N, extracellular Histone H3 adjacent to MPO staining indicative of NETs; BV, blood vessel. **(A,B)** Represent combined data (mean+SD) from “n” biological replicates, **(C)** is representative for “n” biological replicates. ^*^*p* < 0.05 by Mann-Whitney U-test **(A)** or one- way ANOVA followed by Sidak's multiple comparisons correction **(B)**.

## Abbreviations

MFI: mean fluorescence intensity
IMQ: imiquimod
IL: interleukin
PSGL1: P-selectin glycoprotein ligand-1
PLT: platelet
PNCs: Platelet-Neutrophil complexes
PMNs: polymorphonuclear neutrophils
PCA: principal component analysis
PASI: psoriasis area severity index.
