# Supplementary material for: Platelets Aggregate With Neutrophils and Promote Skin Pathology in Psoriasis
Source: Front Immunol. 2019 Aug 16;10:1867. doi: 10.3389/fimmu.2019.01867 (PMC6706802; doi:10.3389/fimmu.2019.01867)
Supplement: Supplementary file 1 [file Table_1.pdf]

**Table S1: Antibodies and staining reagents**

| Item | Fluorophore | Species | Isotype | Company | Product no. |
|---|---|---|---|---|---|
| Isotype control | PE | mouse | IgG1 kappa | eBioscience | 12471442 |
| Isotype control | FITC | mouse | IgM | BioLegend | 401605 |
| Isotype control | APC | mouse | IgG1 kappa | BD Bioscience | 550854 |
| Isotype control | BV421 | mouse | IgG1 kappa | BioLegend | 400157 |
| Isotype control | AF488 | mouse | IgG2b kappa | BioLegend | 402207 |
| Isotype control | AF488 | mouse | IgG1 | BioLegend | 400134 |
| Isotype control | AF647 | mouse | IgM | BioLegend | 401618 |
| Isotype control | PE-Cy7 | mouse | IgG1 | BioLegend | 400126 |
| Anti-hCD15 | PE | mouse | IgG1 kappa | BioLegend | 323006 |
| Anti-hCD66b | FITC | mouse | IgG1 kappa | BioLegend | 305103 |
| Anti-hCD62L | BV421 | mouse | IgG1 kappa | BioLegend | 30482 |
| Anti-hCD3 | AF488 | mouse | IgG1 kappa | BioLegend | 317310 |
| Anti-hCD19 | BV421 | mouse | IgG1 | BioLegend | 302234 |
| Anti-hCD15 | PE-Cy7 | mouse | IgG1 kappa | BioLegend | 323030 |
| Zombie yellow fixable dye | - | - | - | BioLegend | 423103 |
| Anti-hCD41 | PE | mouse | IgG1 | BioLegend | 303706 |
| Anti-hCD61 | PE | mouse | IgG1 | BioLegend | 336406 |
| Anti-hCD66b | AF647 | mouse | IgM | BioLegend | 305109 |
| Anti-hCD62P | AF488 | mouse | IgG1 | BioLegend | 304916 |
| anti-mLy6C | VioBlue | rat | IgG2a κ | Miltenyi | 130-102-929 |
| Anti-mCD45 | APC-Vio770 | rat | IgG2b κ | Miltenyi | 130-118-687 |
| Anti-mCD41 | FITC | rat | IgG1 κ | Miltenyi | 130-102-929 |
| Anti-mLy6G | PE | rat | IgG1 κ | Miltenyi | 130-118-687 |

| | | | | | |
|---|---|---|---|---|---|
| Anti-mCD11b | APC | rat | IgG2b κ | Miltenyi | 130-105-929 |
| Propidium iodide | n/a | - | - | Miltenyi | 130-102-895 |
| TruStain fcX | - | - | - | BioLegend | 130-113-793 |
| Anti-hCD41 | unconjugated | rabbit | IgG1 | 130-093-233 | 130-093-233 |
| Anti-hCD42b | unconjugated | goat | IgG | 422302 | 422302 |
| Anti-hNeutrophil Elastase | unconjugated | mouse | IgG1 | Novus Biologicals | MAB91671-100 |
| Anti-mouse CD41 | unconjugated | rat | IgG1 | GeneTex | GTX-76011 |
| Anti-human/mouse MPO | unconjugated | goat | IgG1 | R&D systems | AF-3667 |
| Anti-histone H3 | unconjugated | rabbit | IgG1 | Novus Biologicals | NB500-171 |
| Anti-rabbit IgG | AF647 | chicken | IgY | ThermoFisher | A-21443 |
| Anti-mouse IgG1 | AF594 | chicken | IgY | ThermoFisher | A-21201 |
| Anti-rat IgG | AF488 | chicken | IgY | ThermoFisher | A-21470 |
| Anti-goat IgG | AF488 | chicken | IgY | ThermoFisher | A-21467 |
| Anti-goat IgG | AF594 | chicken | IgY | ThermoFisher | A-21468 |
