# Supplementary figures and images for: Platelets Aggregate With Neutrophils and Promote Skin Pathology in Psoriasis
Source: Front Immunol. 2019 Aug 16;10:1867. doi: 10.3389/fimmu.2019.01867 (PMC6706802; doi:10.3389/fimmu.2019.01867)

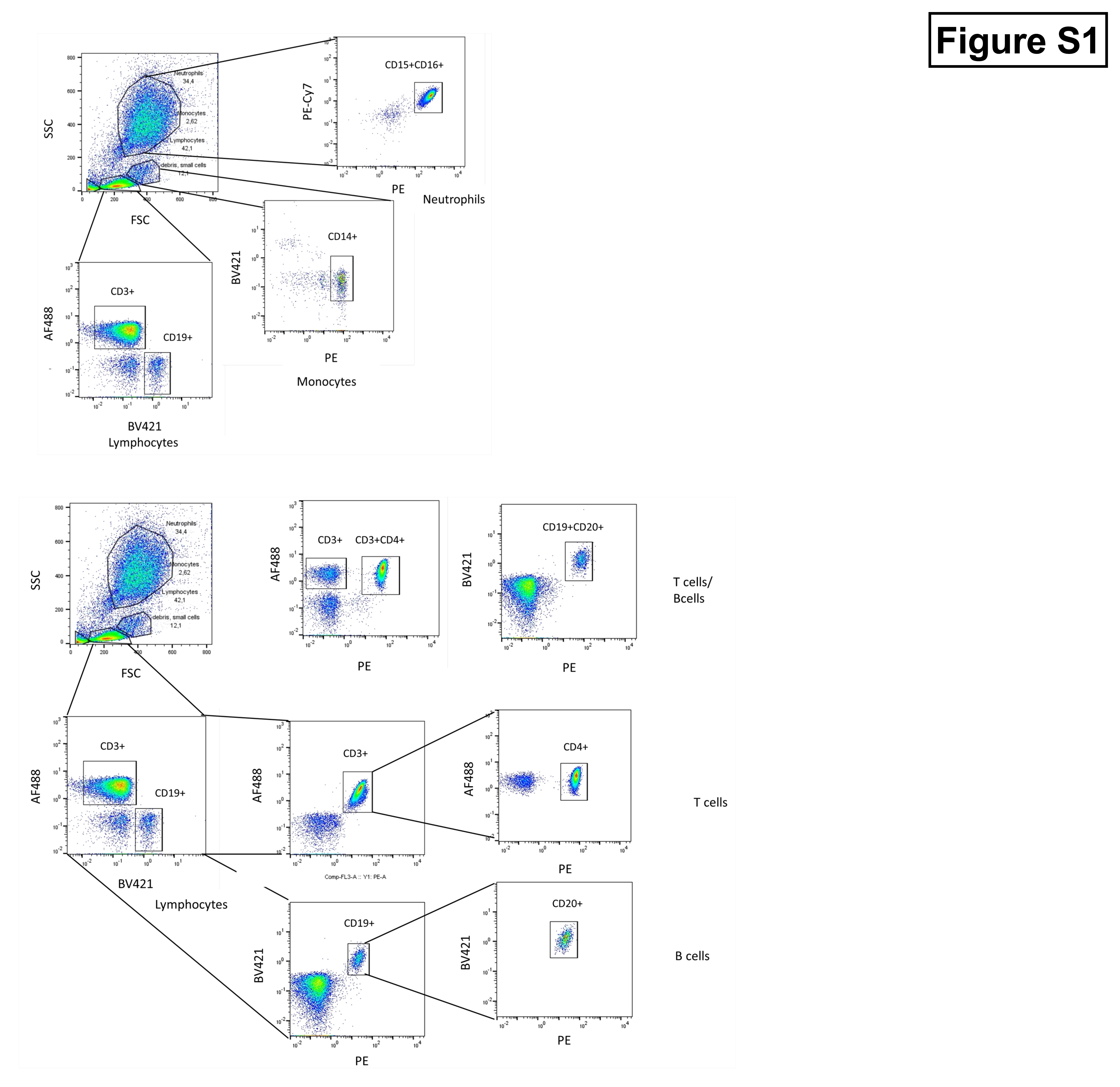

Supplement: Figure S1 — Gating strategy used in the surface antigen screen in human whole blood. [file Image_1.TIF]

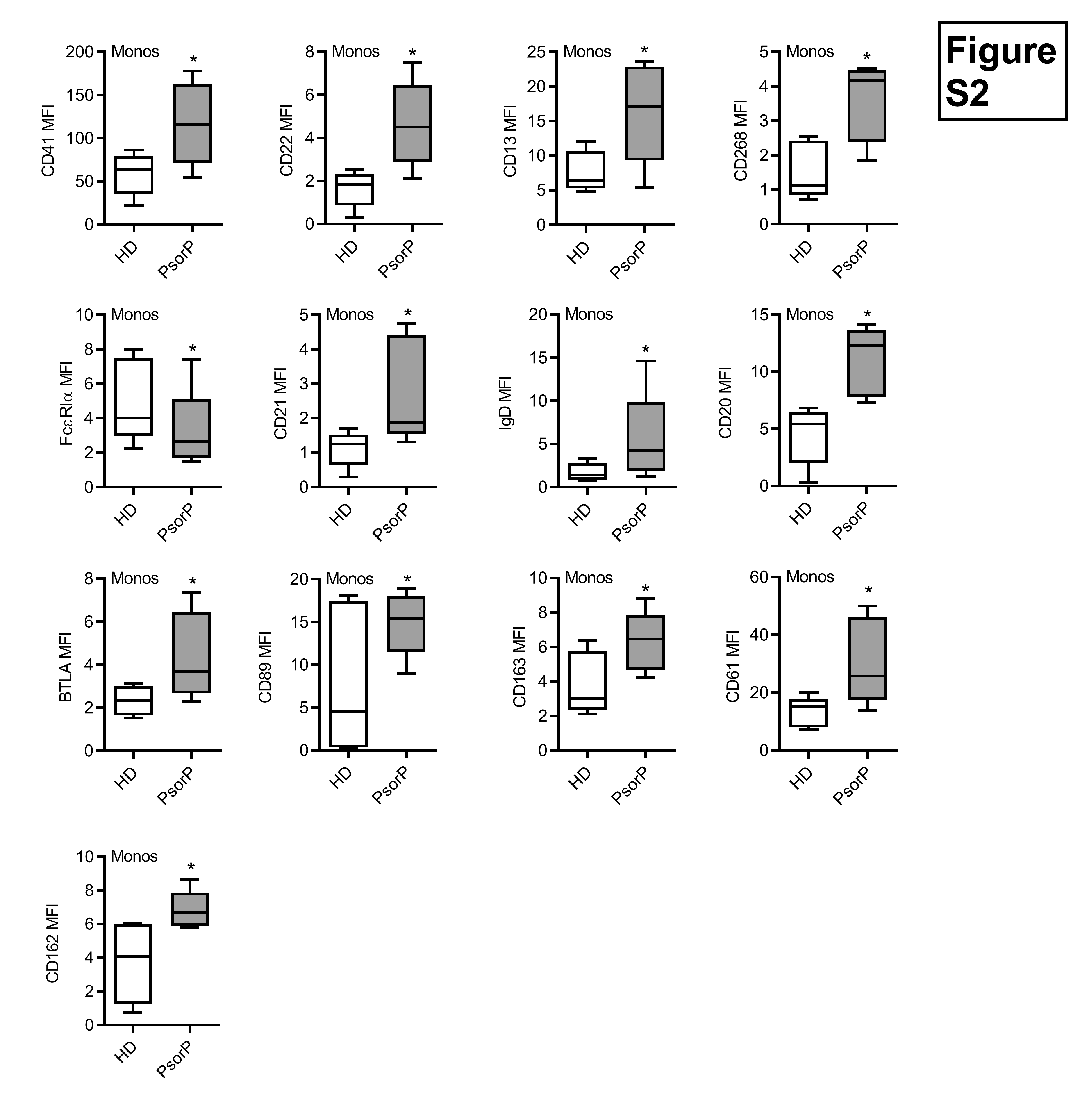

Supplement: Figure S2 — Selected surface antigens with significant differences in MFI between the monocytes from healthy donors (HDs) and psoriasis patients (PsorP), n = 5 each. Graphs represent combined data (mean+SD) from ‘n' biological replicates. *p < 0.1 nominal by two-way ANOVA followed by Tukey's multiple comparisons correction. [file Image_2.TIF]

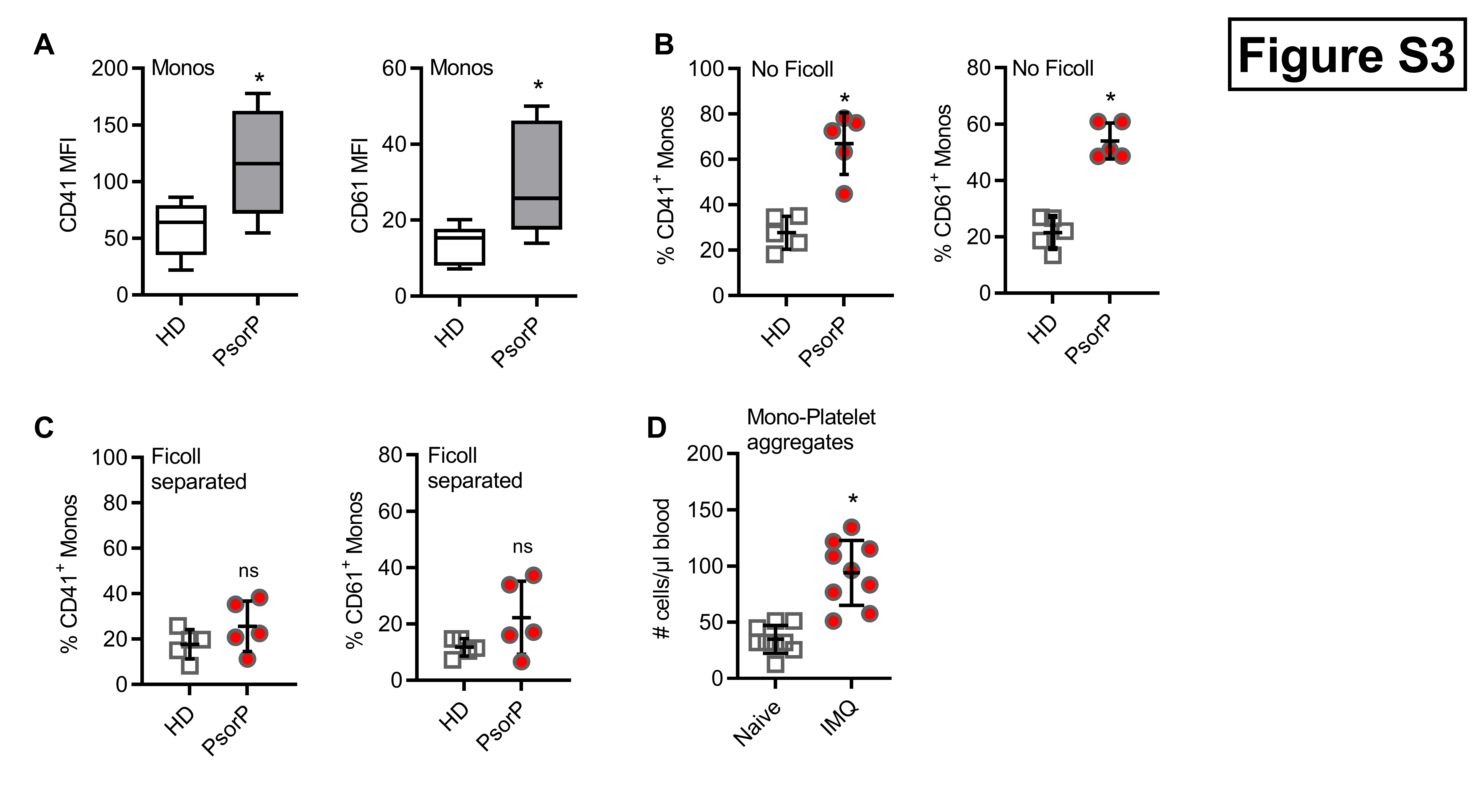

Supplement: Figure S3 — (A) Expression analysis screening results for CD41 (upper) and CD61 (lower panel) MFIs on gated monocytes between healthy blood donors (HDs) and psoriasis patients (PsorP), n = 5 each. (B,C) Flow cytometric analysis of CD41- or CD61-positive gated monocytes in HD or PsorP (HD n = 7, PsorP n = 5) in whole blood samples (B) or Ficoll density gradient centrifugation (C). (D) Mean number of monocyte-platelet aggregates comparing naïve (d0) and IMQ-treated (d5) isotype mice (n = 10 each). (A–D) Represent combined data (mean+SD) from “n” biological replicates (each dot represents one donor). *p < 0.1 nominal by two-way ANOVA in (A–D) *p < 0.05 by unpaired Student's t-tests. [file Image_3.TIF]

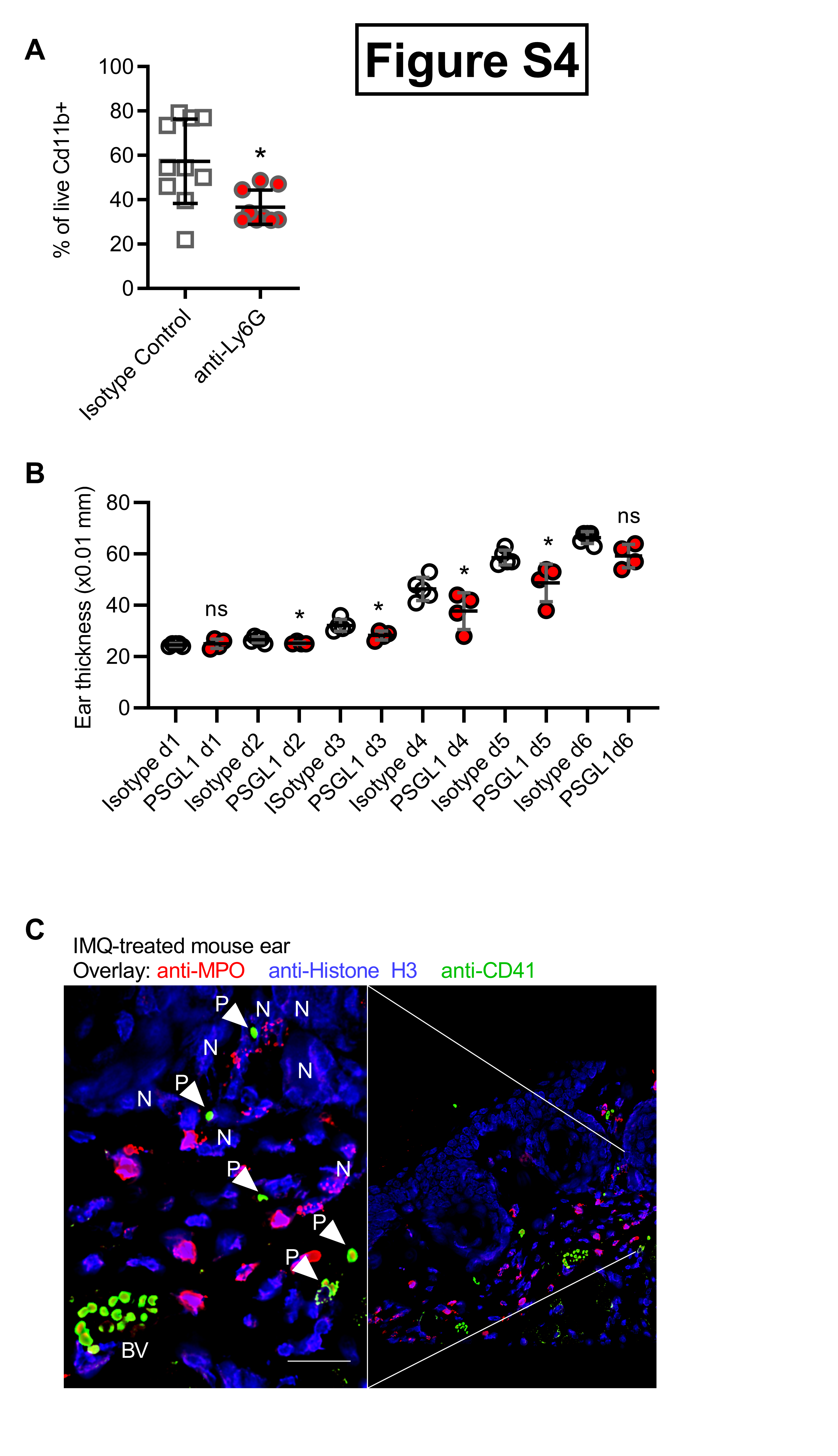

Supplement: Figure S4 — (A) Flow cytometry analysis of PMN depletion on d5 upon infusion of IMQ-treated animals with anti-Ly6G Abs or isotype control (n = 9–10 each). The data represent cells first gated on live cells with PI, then gated on CD11b+ cells, and the data represent Ly6Cint cells as percentage of the CD11b+ population. Cells were isolated from blood at the end of the experiment for both isotype control and anti-Ly6G (clone 1A8) treated mice. (B) Ear thickness (mm × 0.01) measured upon anti-PSGL-1 or control antibody infusion (n = 4–5 each). (C) Representative IF staining from IMQ-treated mouse ear skin (n = 5, scale bar = 20 μm). P, platelet; N, extracellular Histone H3 adjacent to MPO staining indicative of NETs; BV, blood vessel. (A,B) Represent combined data (mean+SD) from “n” biological replicates, (C) is representative for “n” biological replicates. *p < 0.05 by Mann-Whitney U-test (A) or one- way ANOVA followed by Sidak's multiple comparisons correction (B). [file Image_4.TIF]
